# Endoscopic management of esophageal perforations: a multi-center study

**DOI:** 10.1007/s00464-025-12090-9

**Published:** 2025-08-25

**Authors:** Razan Aburumman, Farah Abdul Razzak, Anthony Kerbage, Vitor Brunaldi, Rudy Mrad, Karim Al Annan, Barham Abu Dayyeh

**Affiliations:** 1 Department of Gastroenterology and Hepatology, Mayo Clinic, Rochester, MN USA; 2 Department of Internal Medicine, Henry Ford Hospital, Detroit, MI USA; 3 Department of Internal Medicine, Detroit Medical Center, Detroit, MI USA; 4 Department of Internal Medicine, Cleveland Clinic, Cleveland, OH USA; 5 Department of Anatomy and Surgery, University of São Paulo, Ribeirão Preto, Brazil; 6 Barretos Cancer Hospital, Barretos, Brazil; 7 Department of Internal Medicine, University of Texas Southwestern Medical Center, Dallas, TX USA; 8 Department of Internal Medicine, University of Connecticut, Farmington, CT USA; 9 Division of Gastroenterology and Hepatology, Cedars-Sinai Medical Center, Los Angeles, CA 90048 USA

**Keywords:** Esophageal perforation, Endoscopic pepair, Primary closure, Stent placement

## Abstract

**Background:**

Esophageal perforation (EP) is a rare but life-threatening condition with an incidence of approximately 3.1 per million annually. While iatrogenic injury during endoscopy is the leading cause, other etiologies include spontaneous rupture, trauma, and malignancy. EP can present with nonspecific symptoms, most commonly chest pain or dysphagia, and diagnostic delays are associated with worse outcomes. Multiple non-operative strategies exist, including primary closure, stenting (bypass), combination therapy, and conservative management. However, data guiding the optimal approach remain limited. The aim of this study was to evaluate outcomes of different non-operative management strategies for EP and identify predictors of successful repair.

**Methods:**

We retrospectively analyzed adult patients with EP across three Mayo Clinic sites between 2007 and 2023. Patients were categorized into four groups based on treatment modality. Demographics, clinical features, imaging, endoscopic intervention, and outcomes were recorded. The primary outcome was clinical success, defined as avoidance of surgical intervention. Logistic regression was used to identify predictors of success.

**Results:**

A total of 72 patients were included (mean age 63.7 years, 65.3% male). The most common cause was iatrogenic injury (58.3%), and the distal esophagus was the most frequent site (67.6%). Non-operative success was 100% in the primary closure and combination groups, and 42.9% in the conservative group (p < 0.001). On multivariate analysis, non-conservative therapy significantly predicted success (aOR 22.4, 95% CI [1.2–407.4], p = 0.036).

**Conclusion:**

Primary closure and combination endoscopic approaches offer superior outcomes in managing EP. Early intervention with appropriate modality selection is critical to avoid surgical escalation and improve prognosis.

**Graphical Abstract:**

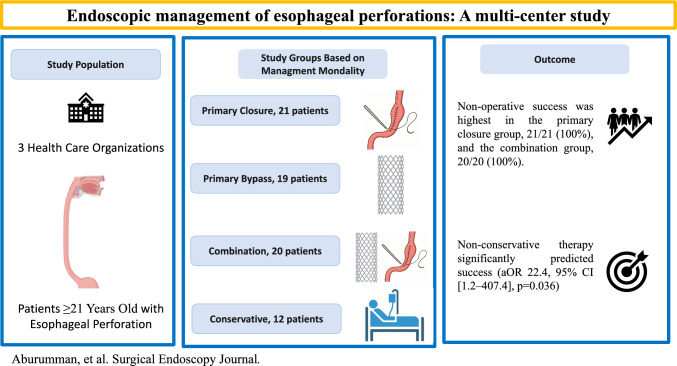

**Supplementary Information:**

The online version contains supplementary material available at 10.1007/s00464-025-12090-9.

Esophageal perforation (EP) is a full-thickness tear of the mucosal and muscular layers of the esophagus [1]. It is a relatively uncommon medical emergency with an incidence of 3.1/1000000 a year [2, 3]. Although its causes are multifactorial, the leading cause worldwide is iatrogenic [4], which often occurs during endoscopic procedures. Other relatively less common causes include spontaneous rupture, ingestion of foreign bodies, trauma, and malignancy [5, 6]. Chest pain is considered the cardinal symptom of EP; however, the clinical presentation varies depending on the underlying cause, location, and duration of rupture [7].

Prompt management of EP is crucial, as the main predictor of complications and survival in those cases is the interval of time from the esophageal injury to its diagnosis and treatment [7–10]. Unfortunately, the initial diagnosis in patients with EPs is incorrect in 50% of cases, and the time interval to diagnosis is longer than 24 h in two-thirds of the cases [11]. There are several possible therapeutic strategies, ranging from medical treatment to the most aggressive, esophagectomy. Somewhere between these two extremes, one finds the mini-invasive techniques, including interventional endoscopy [5]. Available endoscopic management options are clips, esophageal stents, and endoscopic suturing [12]. Although stent placement is the most widely described option, determining the suitable endoscopic closure technique relies on evaluating specific parameters like the defect's location, size, and margins [3, 12].

The debate over the most effective approach to managing EPs persists. Existing evidence guiding the management of EPs relies heavily on retrospective studies from single institutions and a few nationwide studies [2]. The absence of randomized studies underscores the need for a thorough understanding of the factors that influence the success of conservative and endoscopic management. Thus, this study aims to evaluate the outcomes of different EP non-operative management approaches and identify predictors of successful repair.

## Methods

### Study design and patient selection

This study was approved by the Mayo Clinic Institutional Review Board. Patients who underwent an esophagogastroduodenoscopy (EGD) between 2007 and 2023 were retrospectively analyzed through our prospectively registered database across our three Mayo Clinic sites: Rochester, Florida, and Arizona. From this database, we selected adult patients (age ≥ 21) with esophageal perforations. We excluded patients with esophageal fistula, stenosis, and leaks.

Patient baseline information used for this study included age, sex, and co-morbidities (diabetes, hypertension, sleep apnea). Diabetes was classified as either diabetes or no diabetes, regardless of the type, as defined by the American Diabetes Association guidelines for the diagnosis of diabetes [13]. Hypertension was defined as patients who had (1) antihypertensive medications on their medication list at the time of endoscopy or (2) recent outpatient blood pressure documented as > 140 mmHg systolic and/or 90 mmHg diastolic. Sleep apnea was documented based on history and outpatient testing in the electronic medical record.

### Diagnosis

At the time of diagnosis of EP, baseline blood work (lactate, creatinine, and white blood cell count), vital signs, symptoms, level of care, and upper GI series (UGIS) and CT findings were collected. All patients were administered broad-spectrum antibiotics. In the presence of a known perforation serving as a source of inflammation, the inflammatory response syndrome was defined by fulfillment of at least two of the following: temperature > 38 °C or < 36 °C, heart rate > 90, respiratory rate > 20 breaths/minute, and white blood cell count (WBC) of > 12,000/mm or < 4,000 mm [14].

### Non-operative repair

At the time of endoscopic management of EPs, information about the total number of endoscopies, type of perforation, type of non-operative repair employed, stent migration, number of stents placed, time to stent removal, type of stent used, type of clip used, number of sutures, type of suture pattern, failure of non-operative repair, type of operative repair, and duration of hospital stay were collected.

The type of non-operative repair of EPs was categorized into 4 categories, which included primary closure, primary bypass, combination, and conservative, defined as follows:

1. Primary closure: placement of suture and/or clip to close the defect only.
2. Primary bypass: placement of at least one stent across the defect only (with or without suturing the stent to prevent migration)
3. Combination: placement of suture to close the defect in addition to placement of at least one stent (with or without suturing the stent to prevent migration)
4. Conservative: absence of surgical management or endoscopic placement of a stent and/or suture.

The type of stent was based on availability and physician preference. Most stents placed were WallFlex stents (Boston Scientific, Marlborough, MA), however, other stent types were also employed, including: Taewoong (Taewoong Medical, Seoul, South Korea), Alimaxx (Merit Medical Systems, South Jordan, UT), Niti-S stent (Cook Medical, Bloomington, IN), EndoMAXX (Merit Medical Systems, South Jordan, UT), Plastic polyflex (Boston Scientific, Marlborough, MA), and Ultraflex (Boston Scientific, Marlborough, MA).

The suture placement pattern used for primary closure of esophageal perforations was based on the physician’s preference. Most sutures placed were in an interrupted fashion, though others included running, combination of interrupted and running, figure-of-eight, and combination of figure-of-eight and interrupted. Clinical success of non-operative repair was defined as the ability to avoid operative repair.

### Pittsburgh perforation severity score

The Pittsburgh Perforation Severity Score (PPSS) (range, 0–18) was calculated based on 10 clinical variables that are deemed to be important indicators of patient outcomes [15]. Points (1–3) were given to each variable, as documented at the time of diagnosis, according to following scale: 1 = age ≥ 75, tachycardia (> 100 bpm), leukocytosis (> 10,000 WBC/ml), pleural effusion (CT); 2 = fever (> 38.5 °C), uncontained leak (based on CT or barium contrast study), respiratory compromise (respiratory rate > 30), time to diagnosis > 24 h; and 3 = presence of cancer, hypotension. For the purpose of this study, the PPSS was retrospectively calculated and was not solely used to guide management decisions.

### Outcomes

Outcomes included non-operative success, resolution of the perforation based on UGIS/CT scans, length of hospital stay, adverse events, and the mortality rate. Non-operative success was defined as the absence of recurrence of symptoms from the time of diagnosis until the last follow-up. Last follow-up was defined as the last contact with the patient through telecommunications or in clinic, regardless of the reason.

### Statistical analysis

Continuous variables were described by their means and standard deviations (SD) or medians and interquartile ranges (IQR) if the data were skewed. Categorical variables were described by their frequencies. The chi-square test was used to compare frequencies between groups; if expected counts were less than five, then Fisher's exact test was used. Statistical significance was set as *p* < 0.05. One-way ANOVA and Kruskal–Wallis tests were used to compare continuous variables across all treatment types, depending on the normality of the data. A logistic regression was used to determine the predictors of non-operative clinical success, such as treatment modality, age, etiology, location of perforation, and Pittsburgh Perforation Severity Score. The data was imported into IBM SPSS Statistics 28.0, a statistical software used to analyze the data.

### Ethical considerations

This study was conducted in full accordance with the ethical principles of the Declaration of Helsinki and was approved by the Institutional Review Board (IRB) of our institution. Given the retrospective nature of the study, the requirement for informed consent was waived by the IRB. Data were collected from existing records and patient records, and information was anonymized and de-identified prior to analysis.

## Results

### Patient demographics and comorbidities

An initial screening of a prospective database was conducted to identify endoscopic management of esophageal perforations. A total of 72 adult patients with esophageal perforations were identified after esophageal stenosis, fistulas, and leaks were excluded. Out of the 72 patients included in the study, 19 underwent primary bypass (26.4%), 21 patients received primary closure (29.2%), 20 patients were treated with a combination of methods (27.8%), and 12 patients underwent conservative treatment (16.7%). The mean age was 63.7 ± 15.3 years. Among these patients, 65.3% were male. A history of esophageal cancer (EC) was present in 9.7% of the patients, while a history of radiotherapy accounted for 11.1% (Table 1).

**Table 1:** Demographic and clinical characteristics of esophageal perforation patients by treatment modality

Variables	All (*n* = 72)	Primary bypass (*n* = 19)	Primary closure (*n* = 21)	Combination (*n* = 20)	Conservative (*n* = 12)	*p* value
Age (± SD)	63.7 ± 15.3	65.7 ± 12.9	60.1 ± 15.1	66.2 ± 14.7	62.9 ± 20.2	0.584
Male sex (%)	47 (65.3)	12 (63.2)	13 (61.9)	15 (75.0)	7 (58.3)	0.745
History of esophageal cancer (%)	7 (9.7)	3 (15.8)	2 (9.5)	1 (5.0)	1 (8.3)	0.721
History of radiotherapy (%)	8 (11.1)	3 (15.8)	1 (4.8)	2 (10.0)	2 (16.7)	0.642
Diabetes (%)	11 (15.1)	2 (11.1)	2 (8.7)	5 (26.3)	2 (15.4)	0.511
Hypertension (%)	36 (50.0)	11 (57.9)	10 (47.6)	9 (45.0)	6 (50.0)	0.868
Obstructive sleep apnea (%)	10 (13.9)	4 (21.1)	3 (14.3)	3 (15.0)	0 (0.0)	0.428
Etiology of perforation (%) Iatrogenic Spontaneous Boerhaave Foreign body Esophageal cancer Esophageal ulcer	42 (58.3) 8 (11.1) 9 (12.5) 6 (8.3) 2 (2.8) 1 (1.4)	12 (63.2) 1 (5.3) 2 (10.5) 1 (5.3) 2 (10.5) 0 (0)	16 (76.2) 1 (4.8) 2 (9.5) 1 (4.8) 0 (0) 0 (0)	7 (35.0) 3 (15.0) 5 (25.0) 2 (10.0) 0 (0.0) 1 (5.0)	7 (58.3) 3 (25.0) 0 (0.0) 2 (16.7) 0 (0.0) 0 (0.0)	0.224
Site of perforation (%) Proximal Middle Distal	11 (15.5) 12 (16.9) 48 (67.6)	1 (5.3)^a^ 3 (15.8) 15 (78.9)	0 (0.0)^a^ 7 (33.3) 14 (66.7)	3 (15.8)^a,b^ 2 (10.5) 14 (73.7)	7 (58.3)^b^ 0 (0.0) 5 (41.7)	** < 0.001**
Size of perforation (mm ± SD)	17.6 ± 12.6	20.7 ± 13.7	13.6 ± 13.3	19.5 ± 10.3	15.6 ± 21.3	0.606
Symptoms at diagnosis (%) Dysphagia Chest pain Abdominal pain Fever Other	20 (27.8) 16 (22.2) 1 (1.4) 3 (4.2) 27 (37.5)	6 (31.6) 6 (31.6) 0 (0.0) 1 (5.3) 7 (36.8)	4 (19.0) 4 (19.0) 1 (4.8) 0 (0) 7 (33.3)	5 (25.0) 5 (25.0) 0 (0) 2 (10.0) 7 (35.0)	5 (41.7) 1 (8.3) 0 (0) 0 (0) 6 (50.0)	0.539 0.473 0.482 0.362 0.798
Pittsburgh perforation severity score (median—IQR)^Δ^	2 (1–4)	4 (1–6)^a^	1 (1–2)^b^	3 (1.5–4)^a,b^	3 (0.75–4.25)^a,b^	**0.037**
SIRS (%)	32 (44.4)	9 (47.4)	7 (33.3)	11 (55.0)	5 (41.7)	0.561
Time to diagnosis (median days, IQR)^Δ^	0 (0–0.5)	0 (0–0.5)	0 (0–0.38)	0 (0–0.4)	0 (0–4)	0.632
Time from diagnosis to treatment (median days, IQR)^Δ^	0.5 (0–1.5)	0.5 (0–2)	0.5 (0–1.8)	0.8 (0.5–3)	0 (0–1)	0.133

Bold value indicate that it is statistically significant *p* value <0.05

*SIRS* Systemic inflammatory response syndrome, *IQR* Interquartile Range

^a,b^significant difference on pairwise comparisons

^Δ^Kruskal–Wallis test used (skewed data)

### Perforation characteristics and clinical presentation

The etiology of perforations was mostly iatrogenic (58.3%), followed by Boerhaave syndrome (12.5%). The most common site of perforation was the distal esophagus (67.6%), however, when comparing different treatment groups, patients treated with conservative treatment were more likely to have a perforation in the proximal esophagus (53.8%) compared to patients treated with primary bypass or primary closure. The mean size of the perforations was 17.6 ± 12.6 mm, largest on average in the primary bypass group (20.7 mm ± 13.7) and smallest in the primary closure group (13.6 mm ± 13.3). The most common symptoms at diagnosis were dysphagia (27.8%) and chest pain (22.2%). The Pittsburgh Perforation Severity Score had a median of 2 (IQR 1–4), and SIRS was present in 44.4% of the patients. The time from symptom onset to diagnosis had a median of 0 days, and the time from diagnosis to treatment had a median of 0.5 days.

### Outcomes by treatment group

**Fig. 1:**
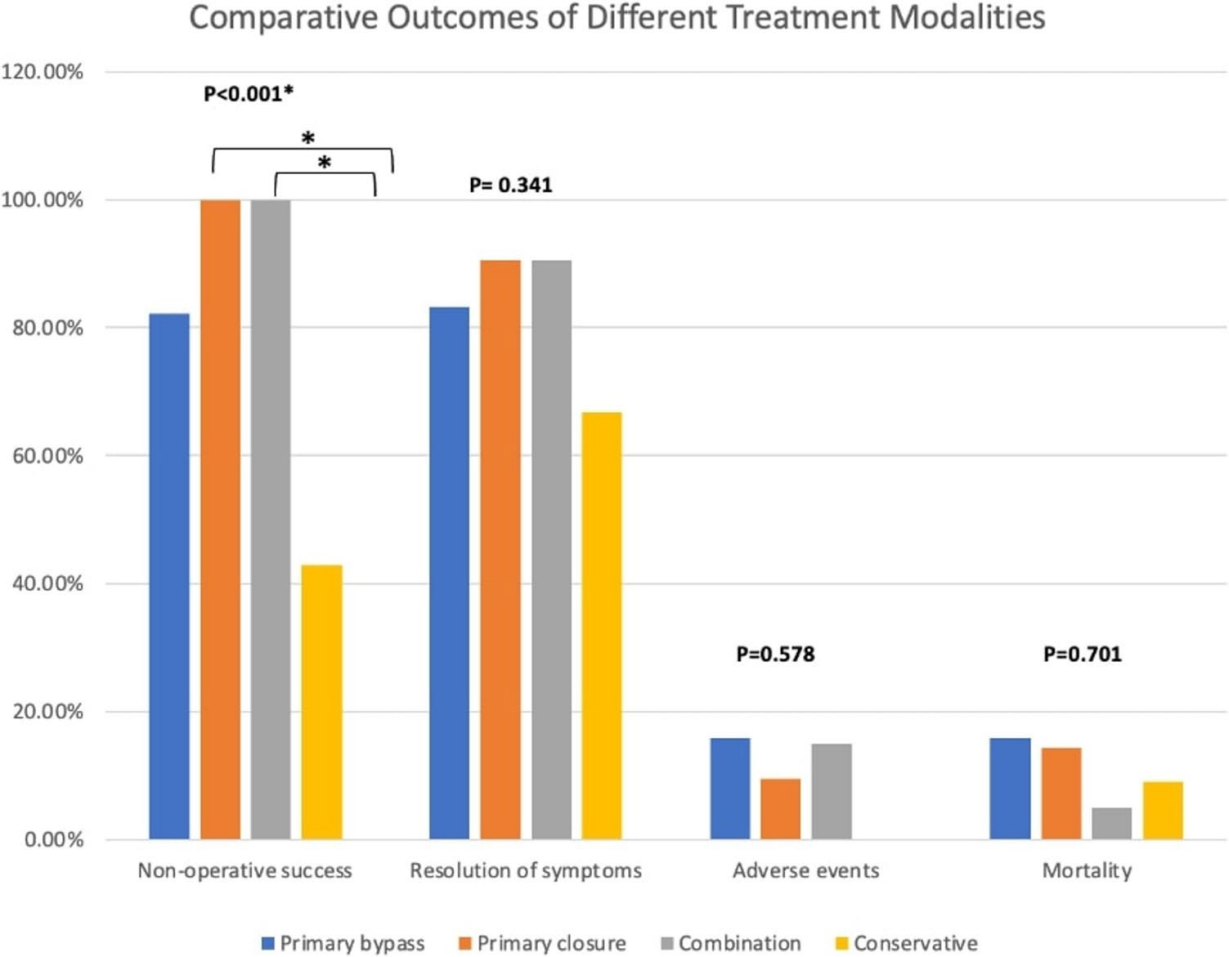
Comparative outcomes of different treatment modalities in managing esophageal perforations. The bar chart compares four approaches—primary bypass (blue), primary closure (orange), combination therapy (gray), and conservative management (yellow)—across key clinical outcomes: non-operative success, resolution of symptoms, adverse events, and mortality. Asterisks (*) indicate statistically significant comparisons

Outcomes of different treatments are shown in Fig. 1. Non-operative success was highest in the primary closure group (100%) and combination group (100%), and lowest in the conservative group (42.9%), with the difference being statistically significant (p < 0.001). Resolution based on UGIS/CT was higher in primary bypass, primary closure, and combination treatment groups (83.3%, 90.5%, and 90.5%, respectively), and compared to the conservative group (66.7%), but these differences were not statistically significant (p = 0.341). The median length of hospital stay varied, with the primary closure group having the shortest median stay (6 days) and the primary bypass group the longest (14 days), approaching statistical significance (p = 0.053). Adverse events and mortality rates were relatively low across all treatments and were not significantly different between treatment groups (p = 0.578 and p = 0.701, respectively). Adverse events were reported in 8 cases (11.4%), with 4 incidents related to the perforation itself (including 3 cases of mediastinal abscesses and 1 case of septic shock) and 4 therapy-related adverse events (1 stent-related pain, 1 stent migration, 1 stent erosion with esophageal ulceration and bleeding, and 1 significant tissue ingrowth through the stent). Mortality at the end of follow-up was 8 cases (11.3%), with 4 (5.6%) of the deaths unrelated to the perforation or therapy, while the other 4 (5.6%) were directly attributable to the perforation, with 2 (2.8%) cases of respiratory failure and 2 (2.8%) cases of septic shock with multiorgan failure.

Notably, the overall rate of avoiding operative intervention was 91.7%, with only 6 patients (8.3%) ultimately requiring surgery following failure of initial non-operative management. This included 1 patient from the primary closure group (1/21; 4.8%), 2 from the primary bypass group (2/19; 10.5%), 2 from the combination therapy group (2/20; 10.0%), and 1 from the conservative group (1/12; 8.3%). There were no mortalities among these patients. Given the limited number of surgical cases, a detailed analysis of surgical outcomes was not performed.

### Predictors of Successful Clinical Outcome

Logistic regression analysis was conducted to assess the predictors of non-operative success. Treatment modalities were dichotomized into conservative and non-conservative therapy, because non-conservative methods (including primary bypass, primary closure, and combination treatment) were significantly associated with non-operative success on bivariate analysis. Other covariates incorporated into the model were the Pittsburgh Perforation Severity Score, the etiology of perforation, and the location of perforation. The analysis showed that non-conservative therapy is a predictor of non-operative success with a p-value of 0.036 and an adjusted odds ratio of 22.4 (95% CI 1.2–407.4) (Supplementary Table 1).

## Discussion

In recent years, conservative and endoscopic approaches have become popular for managing EPs. This retrospective study aimed to report our 17-year experience with this life-threatening condition across three sites, and compare different endoscopic methods and the conservative approach in the management of EP.

In our cohort, the mean age was 63.7 ± 15.3 years, which is notably higher than that reported in previous studies. A systematic review of 1,319 studies on acute esophageal perforations, encompassing 2,830 cases, found an average patient age of 58.4 years [16]. Regarding gender, studies have consistently shown that this condition is more prevalent in males [17–19], and 65.3% of our sample were males. The most common etiology of esophageal perforation in our cohort was iatrogenic, accounting for 58.3% of cases. This is consistent with prior studies that identified iatrogenic factors as responsible for up to 59% of esophageal perforations [9, 20]. In our population, the most common site of perforation was the distal esophagus (67.6%). This finding aligns with some studies [21], though the predominant site of esophageal perforation remains a topic of debate.

Patients with EP can present with a variety of symptoms, making diagnosis challenging and requiring a high index of suspicion. Notably, a missed diagnosis that was first made at autopsy has been reported in 17% of cases [15]. In our study population, the most common symptoms at diagnosis were dysphagia (27.8%) and chest pain (22.2%), both of which are nonspecific and can stem from a wide variety of conditions. Notably, the SIRS criteria were met at the time of presentation in 44.4% of our patients, which is considerably higher than the 23.3% reported in a systematic review of 898 patients with EP [16]. Despite these challenges, we found that the median time from symptom onset to diagnosis was 0 days, and the median time from diagnosis to treatment was only 0.5 days. This prompt response is critical, as delays can significantly increase the risk of serious complications, including sepsis, mediastinitis, multiorgan failure, and even mortality [8, 10, 20, 21].

In our cohort, when comparing different treatment methods, the conservative group showed the lowest non-operative success rate at 42.9%, while both the primary closure and combination groups achieved 100% success, a difference that was statistically significant (p < 0.001). There were no significant differences among the groups concerning symptom resolution or length of hospital stay. The median lengths of stay varied, with the primary closure group having the shortest at 6 days and the primary bypass group the longest at 14 days. Notably, both of these lengths are lower than the mean hospital stay of 32.9 days reported in a systematic review of 2,971 patients with esophageal perforation treated through various surgical and non-surgical methods [22].

Esophageal perforation presents high morbidity and mortality, with mortality rates ranging anywhere from 4 to 50% [8, 23]. In our study, adverse events and mortality rates were relatively low across all treatment groups, showing no significant differences (p = 0.578 and p = 0.701, respectively). By the end of the follow-up period, our cohort experienced an overall mortality rate of 11% (8 cases), with 4 of those deaths directly attributed to the perforation.

### Limitations

This study has several notable limitations that should be considered when interpreting the results. First, its retrospective design inherently carries the risk of selection bias, as patients were identified from a database and may not represent the entire population of individuals with esophageal perforations. Additionally, we acknowledge that reporting time to diagnosis and intervention in hours would offer greater precision; however, the retrospective nature of the data and lack of uniform timestamp documentation limited reporting to calendar days. Also, the relatively small sample size limits the ability to draw broad conclusions, reduces the statistical power to detect significant differences between treatment groups, and widens the confidence interval range for our results. Future prospective multicenter studies with larger cohorts are needed to validate these observations and explore subgroup-specific outcomes in greater detail. Variability in treatment protocols and physician preferences across different institutions could introduce inconsistencies in the data. Lastly, the reliance on clinical assessments and imaging interpretations can also lead to variability in diagnosis and management decisions.

## Conclusion

In conclusion, this study underscores the effectiveness of various non-operative management strategies for esophageal perforations. We showed that treatment modality is a significant predictor of success, particularly highlighting the superior outcomes associated with primary closure and combination techniques. Future prospective studies are essential to validate these results and to develop standardized treatment protocols that can further enhance patient care. Ultimately, a deeper understanding of the factors influencing outcomes in esophageal perforation management could lead to improved survival rates and quality of life for affected patients.

## Supplementary Information

Below is the link to the electronic supplementary material.

Supplementary file1 (DOCX 15 KB)

## References

[1] DeVivo A et al (2022) High risk and low prevalence diseases: Esophageal perforation. Am J Emerg Med 53:29–36 34971919 10.1016/j.ajem.2021.12.017

[2] Søreide JA, Viste A (2011) Esophageal perforation: diagnostic work-up and clinical decision-making in the first 24 hours. Scand J Trauma Resusc Emerg Med 19:66 22035338 10.1186/1757-7241-19-66 PMC3219576

[3] Fairbairn K, Worrell SG (2023) Esophageal perforation: is surgery still necessary? Thorac Surg Clin 33(2):117–123 37045480 10.1016/j.thorsurg.2023.01.005

[4] Nirula R (2014) Esophageal perforation. Surg Clin North Am 94(1):35–41 24267495 10.1016/j.suc.2013.10.003

[5] Chirica M et al (2010) Esophageal perforations. J Visc Surg 147(3):e117–e128 20833121 10.1016/j.jviscsurg.2010.08.003

[6] Watkins JR, Farivar AS (2018) Endoluminal therapies for esophageal perforations and leaks. Thorac Surg Clin 28(4):541–554 30268300 10.1016/j.thorsurg.2018.07.002

[7] Khaitan PG, Famiglietti A, Watson TJ (2022) The etiology, diagnosis, and management of esophageal perforation. J Gastrointest Surg 26(12):2606–2615 36138308 10.1007/s11605-022-05454-2

[8] Deng Y et al (2021) Current treatment and outcome of esophageal perforation: a single-center experience and a pooled analysis. Medicine (Baltimore) 100(16):e25600 33879724 10.1097/MD.0000000000025600 PMC8078246

[9] Brinster CJ et al (2004) Evolving options in the management of esophageal perforation. Ann Thorac Surg 77(4):1475–1483 15063302 10.1016/j.athoracsur.2003.08.037

[10] García-Moreno V et al (2022) Treatment of esophageal perforation: a review of our experience at a tertiary referral hospital spanning the past 19 years. Rev Gastroenterol Méx (Engl Ed) 87(4):405–410 34887217 10.1016/j.rgmxen.2021.11.014

[11] Griffiths EA et al (2009) Thirty-four cases of esophageal perforation: the experience of a district general hospital in the UK. Dis Esophagus 22(7):616–625 19302220 10.1111/j.1442-2050.2009.00959.x

[12] Gurwara S, Clayton S (2019) Esophageal perforations: an endoscopic approach to management. Curr Gastroenterol Rep 21(11):57 31749030 10.1007/s11894-019-0730-5

[13] (n.a.) (2019) Standards of medical care in diabetes-2019. Diabetes Care 42(Suppl 1):S13-s28 10.2337/dc19-S002 30559228

[14] Baddam S, Burns B (2025) Systemic inflammatory response syndrome. In: StatPearls. Treasure Island, FL: StatPearls Publishing 31613449

[15] Moletta L et al (2022) Could the Pittsburgh Severity Score guide the treatment of esophageal perforation? Experience of a single referral center. J Trauma Acute Care Surg 92(1):108–116 34561399 10.1097/TA.0000000000003417

[16] Sdralis EIK et al (2017) Epidemiology, diagnosis, and management of esophageal perforations: systematic review. Dis Esophagus 30(8):1–6 28575240 10.1093/dote/dox013

[17] Edholm D, Andersson RE, Frankel A (2022) Esophageal perforations: a population-based nationwide study in Sweden with survival analysis. Scand J Gastroenterol 57(9):1018–1023 35400263 10.1080/00365521.2022.2060051

[18] Shahriarirad R et al (2023) Esophageal perforation etiology, outcome, and the role of surgical management – an 18-year experience of surgical cases in a referral center. BMC Surg 23(1):177 37370071 10.1186/s12893-023-02080-w PMC10304640

[19] Cross MR, Greenwald MF, Dahhan A (2015) Esophageal perforation and acute bacterial mediastinitis: other causes of chest pain that can be easily missed. Medicine (Baltimore). 10.1097/MD.0000000000001232 26266352 10.1097/MD.0000000000001232 PMC4616702

[20] Kassem MM, Wallen JM (2024) Esophageal peforation and tears. In: StatPearls. Treasure Island, FL: StatPearls Publishing 30335331

[21] Zimmermann M et al (2017) Predictors of morbidity and mortality in esophageal perforation: retrospective study of 80 patients. Scand J Surg 106(2):126–132 27334795 10.1177/1457496916654097

[22] Vidarsdottir H et al (2010) Oesophageal perforations in Iceland: a whole population study on incidence, aetiology and surgical outcome. Thorac Cardiovasc Surg 58(8):476–480 21110271 10.1055/s-0030-1250347

[23] Biancari F et al (2013) Current treatment and outcome of esophageal perforations in adults: systematic review and meta-analysis of 75 studies. World J Surg 37(5):1051–1059 23440483 10.1007/s00268-013-1951-7

[24] Kaman L et al (2010) Management of esophageal perforation in adults. Gastroenterol Res 3(6):235–244 10.4021/gr263w PMC5139851 27942303

