# Supplementary material for: Endoscopic management of esophageal perforations: a multi-center study
Source: Surg Endosc. 2025 Aug 25;39(10):6719–25. doi: 10.1007/s00464-025-12090-9 (PMC12500714; doi:10.1007/s00464-025-12090-9)
Supplement: Supplementary file 1 — Supplementary file1 (DOCX 15 KB) [file 464_2025_12090_MOESM1_ESM.docx]

Table 1: Logistic regression model variables

| **Predictors** | **Odds Ratio** | **95% CI**  **Lower Upper** | | **P- Value** |
| --- | --- | --- | --- | --- |
| **Conservation management** | 22.356 | 1.227 | 407.423 | **0.036** |
| **Pittsburgh Perforation Severity Score** | 1.117 | 0.581 | 2.151 | 0.740 |
| **Iatrogenic perforation** |  |  |  | 0.992 |
| **Spontaneous perforation** | 0.000 | 0.000 |  | 1.000 |
| **Boerhaave’s syndrome** | 0.000 | 0.000 |  | 1.000 |
| **Foreign body or food impaction** | 1.107 | 0.000 |  | 1.000 |
| **Postoperative leak or perforation** | 0.000 | 0.000 |  | 1.000 |
| **Malignant perforation** | 1.126 | 0.000 |  | 1.000 |
| **Esophageal ulcer** | 0.327 | 0.000 |  | 1.000 |
| **Proximal perforation** |  |  |  | 0.991 |
| **Middle perforation** | 89676554.77 | 0.000 |  | 0.999 |
| **Distal perforation** | 46341256.63 |  |  | 1.000 |
